# Assisted Parkinsonism Diagnosis Using Multimodal MRI—The Role of Clinical Insights

**DOI:** 10.1002/brb3.70274

**Published:** 2025-01-19

**Authors:** Tobias Meindl, Alexander Hapfelmeier, Tobias Mantel, Angela Jochim, Jonas Deppe, Silke Zwirner, Jan S. Kirschke, Yong Li, Bernhard Haslinger

**Affiliations:** ^1^ Department of Neurology, Klinikum rechts der Isar Technical University of Munich Munich Germany; ^2^ Institute of AI and Informatics in Medicine, School of Medicine Technical University of Munich Munich Germany; ^3^ Institute of General Practice and Health Services Research, School of Medicine Technical University of Munich Munich Germany; ^4^ Department of Diagnostic and Interventional Neuroradiology, Klinikum rechts der Isar Technical University Munich Munich Germany

**Keywords:** automated diagnosis, decision support, MRI, multiple system atrophy, Parkinson's disease, progressive supranuclear palsy

## Abstract

**Background:**

While automated methods for differential diagnosis of parkinsonian syndromes based on MRI imaging have been introduced, their implementation in clinical practice still underlies considerable challenges.

**Objective:**

To assess whether the performance of classifiers based on imaging derived biomarkers is improved with the addition of basic clinical information and to provide a practical solution to address the insecurity of classification results due to the uncertain clinical diagnosis they are based on.

**Methods:**

Retro‐ and prospectively collected data from multimodal MRI and standardized clinical datasets of 229 patients with PD (*n* = 167), PSP (*n* = 44), or MSA (*n* = 18) underwent multinomial classification in a benchmark study comparing the performance of nine machine learning methods. A predictor space of imaging variables, either with or without clinical information, was investigated. Classification results were assessed using multiclass AUCs. Individual predicted probabilities were visualized to address diagnostic uncertainty.

**Results:**

Clinical diagnosis was accurately confirmed using machine learning models with only small differences when using imaging and clinical signs versus imaging variables only (expected multiclass AUC of 0.95 vs. 0.92). Still, multinomial classification is hampered by imbalanced class frequencies. The most discriminatory variables were responsiveness to levodopa, vertical gaze palsy, and the volumes of subcortical structures, including the red nucleus.

**Conclusion:**

Machine‐learning‐assisted classification of MR‐imaging biomarkers gathered in routine care can assist in the diagnosis of parkinsonian syndromes as part of the diagnostic workup. We provide a visual method that aids the interpretation of neuroimaging‐based classification results of the three main parkinsonian syndromes, improving clinical interpretability.

## Graphical abstract

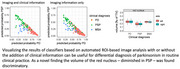

## 1 Introduction

Primary parkinsonian syndromes comprise idiopathic Parkinson's disease (PD) and atypical parkinsonian syndromes (aPS). The latter comprises progressive supranuclear palsy (PSP), multiple system atrophy (MSA), and corticobasal syndrome (CBS). Establishing a diagnosis of sufficient certainty is important for several reasons: It is relevant to confidently counsel patients about the likely clinical course and response to therapy of their disease. Regarding clinical trials, sample sizes could be reduced if a higher fraction of patients truly suffering from their expected diagnosis could be enrolled. However, the accuracy of diagnosis based on current criteria, even with adequate follow‐up, does not exceed 85% (Ali et al. 2019; Koga et al. 2015; Rizzo et al. 2016).

To increase diagnostic certainty, it is straightforward to extract maximum information from MR imaging as it is widely available and routinely applied in parkinsonian syndromes. MR imaging markers have been assessed in several studies. Early conventional signs like the hummingbird and morning glory signs showed good specificity but poor sensitivity (Mueller et al. 2018). While this was improved using quantitative measures such as the midsagittal midbrain or pons (Hussl et al. 2010; Mangesius et al. 2018) and combined indices from these measures (Quattrone et al. 2018, 2008), the drawbacks of manual analysis and potential human inference remained. As a consequence, automated diagnostic tools with machine‐learning (ML) approaches are being developed to distinguish between PD and aPS using information gained in T1 anatomical and/or DTI imaging, differentiating clinically‐defined diagnoses of PD and MSA (Beliveau et al. 2021; Krismer et al. 2019); PD and PSP (Potrusil et al. 2020; Seki et al. 2018); and PD, PSP, and MSA (Archer et al. 2019; Chougar et al. 2021; Huppertz et al. 2016; Scherfler et al. 2016).

Clinically, the suspicion of an atypical Parkinson's syndrome is usually aroused by the presence of certain signs, often called potential “red flags,” leading to the consultation of movement disorders experts and potentially further diagnostic workup. Regarding the potentially supporting role of neuroimaging‐based classifications in clinical practice, it remains an interesting question of how much the automated interpretation of imaging could contribute to the emergence of such a suspicion outside of expert centers compared to relying on basic clinical findings only. To elucidate this aspect, the present study evaluated the separability of PD, PSP, and MSA in classification approaches that either combined easily obtainable clinical information (encompassing potential “red flags”) and automatically processed multimodal imaging or relied on imaging only. Therein, our benchmark study investigated nine ML models able to deal with multinomial outcomes to compare how well they handle multiple differential diagnoses, mirroring the challenge in daily clinical practice. Results were explored to determine which cerebral structures contribute to differentiation.

In addition, we offer a visual representation of predicted probabilities that could be useful for clinical applicability. This helps gauge how well a patient's suspected diagnosis aligns with the expected diagnosis among similar patients, serving as a practical way to tackle diagnostic uncertainty.

## 2 Methods

### 2.1 Subjects and Scans

We included a total of 229 patients with a diagnosis of PD, PSP, or MSA who received an MRI scan in our hospital between 2011 and 2019. Thirty‐six patients were included retrospectively and 193 prospectively. The current diagnostic criteria of the Movement Disorders Society for PD (Postuma et al. 2015) and PSP (Hoglinger et al. 2017) and the revised AAN consensus criteria for MSA (Gilman et al. 2008) were applied for clinical diagnosis. For study inclusion, participants had to fulfill the respective two highest degrees of clinical diagnostic certainty for their respective diagnoses that are labeled “clinically established” or “clinically probable” in the case of PD or “probable” and “possible” in the case of MSA and PSP (as determined by the respective publications). All disease‐specific phenotypes were included. Patients’ characteristics are presented in Table 1.

**TABLE 1:** Patient characteristics.

	PD	PSP	MSA
*n* Patients	167	44	18
Certainty of diagnosis (patients)			
	Clinically established: 75	Probable: 41	Probable: 13
	Clinically probable: 92	Possible: 3	Possible: 5
Clinical subtypes (patients)			
	PD hypokinetic: 96	PSP‐RS: 35	MSA‐P: 12
	PD tremor‐dominant: 42	PSP‐P: 5	MSA‐C: 6
	PD mixed: 29	PSP‐SL: 1	
		PSP‐F: 1	
		PSP‐CBS: 1	
		PSP‐OM: 1	
Demographics			
Age, mean ± SD	65.42 ± 10.45	70.59 ± 7.60	58.94 ± 7.18
Sex, m/f:	112/55	24/20	14/4
Disease duration	7.03 ± 6.20 (14)	2.83 ± 2.46 (2)	3.13 ± 2.75 (3)
Disease duration < 2/2–4/4–7/> 7 years	30/26/30/67 (14)	14/7/8/3 (2)	4/7/2/2 (3)
H&Y‐stage	2.25 ± 1.05 (17)	3.10 ± 1.08 (4)	3.59 ± 1.37 (1)
MoCA	24.31 ± 4.85 (39)	22.34 ± 4.63 (9)	24.92 ± 4.29 (5)
CES‐D	14.79 ± 9.12 (55)	14.20 ± 9.63 (14)	18.10 ± 7.92 (8)
NMS‐Questionnaire	8.21 ± 5.34 (38)	9.06 ± 5.44 (10)	11.93 ± 2.43 (4)

*Note*: Aggregate statistics are presented as mean ± SD, the number of missing values for a parameter is shown in parenthesis.

### 2.2 Ethics Approval

The study has been approved by the local ethics review board (Ethikkommission der Technischen Universität München). Patients whose data were not only acquired retrospectively gave their written informed consent before entering the prospective phase of the study. Informed consent was waived from patients whose data was used only retrospectively.

### 2.3 Clinical Examination

Prospectively included patients who received a standardized physical examination and history. When retrospectively included, the relevant information was reconstructed from medical notes to the greatest possible extent. The following clinical items were used for ML as binary variables (presence yes/no): symmetry of symptoms, responsiveness to l‐DOPA, presence of rigor, bradykinesia, rest tremor, postural tremor, kinetic tremor, postural instability, vertical gaze palsy, pyramidal tract signs, cerebellar signs, dystonia, frontal signs, as well as the anamnestic complaint about orthostatic hypotension. None of the symptoms or signs were formally defined, and the statement on the presence was left to the discretion of the treating physician. This best represents clinical practice where patients present mainly to non‐movement disorders expert neurologists.

### 2.4 Scanner and Sequences

All scans were acquired between 2011 and 2019 on a Philips‐Achieva 3T Scanner. As the initial scanner was replaced in October 2017, two different scanners were used in the end. One hundred forty imaging sessions were acquired on Scanner 1 (Achieva, software 5.1, 8 channel head coil) and 89 scans on Scanner 2 (Achieva d‐stream, software 5.4, 32 channel head coil). A standardized protocol consisting of T1, FLAIR, T2, DTI (6 gradient directions to minimize total scanning time) and SWI sequences were used. Details of the sequence parameters, which were slightly optimized over the acquisition period, are stated in Table S1.

### 2.5 Image Processing

Anatomical images were processed using the Computational Anatomy Toolbox (CAT12 r1152, http://www.neuro.uni‐jena.de/cat/), FreeSurfer v. 6.0 (https://surfer.nmr.mgh.harvard.edu/), and MIST (Multimodal Image Segmentation Tool, https://fsl.fmrib.ox.ac.uk/fsl/fslwiki/MIST/). DTI scans were preprocessed using ExploreDTI (v. 4.8.6, www.exploredti.com) and Statistic Parametric Mapping (SPM r7219, https://www.fil.ion.ucl.ac.uk/spm/).

The goal of image processing was to obtain regions of interest (ROIs)‐based metrics from different imaging modalities. The workflow consisted of a combination of standard procedures with default parameters. ROIs obtained were: (i) supratentorial (cortical) structures defined by the Destrieux Atlas of gray and white matter ROIs (Destrieux et al. 2010); (ii) infratentorial structures comprising those derived from FreeSurfer's standard subcortical and brainstem analysis streams (Fischl et al. 2002; Iglesias et al. 2015) as well as the red nucleus, substantia nigra, and subthalamic nucleus segmented using MIST (Visser et al. 2016), and the middle cerebellar peduncle (as defined in the JHU white matter atlas; Mori et al. 2008) using CAT12. A detailed description is presented in the supplementary methods (including Figures S1 and S2).

The metrics used for classification were normalized volumes of each ROI (ROI volume/total intracranial volume [TIV]) and the mean and standard deviation of the fractional anisotropy (FA) and mean diffusivity (MD) values within each ROI. For cortical ROIs, additionally, the mean and standard deviation of the cortical thickness as well as the surface area were obtained. Furthermore, aggregated lobar volumes were calculated, summing up the respective cortical grey matter ROIs. For bilateral structures, the minimum, the sum, and the absolute and relative difference between the left‐ and right‐sided values of the structure were calculated. In total, 2518 imaging‐derived metrics were obtained.

### 2.6 Machine Learning

Analysis was done using R 3.6.1 and specifically relied on the package “mlr” for ML (Bischl et al. 2016; R Core Team 2020). ML was used to predict the clinical diagnosis (“PD,” “PSP,” “MSA”) using age, sex, disease duration, imaging metrics including (Approach 1) and excluding (Approach 2) the clinical parameters mentioned above.

In a preliminary filtering step, the predictor variables (imaging and clinical metrics) were reduced to 500 variables with the highest entropy‐based FSelector's information gain (Romanski, Kotthoff, and Schratz 2021). Then, nine models were applied, namely AdaBoost.M1 regression trees, a conditional inference regression tree, a conditional inference random forest, a C4.5 regression tree, a C5.0 regression tree, a Naïve Bayes classifier, a PART decision list, a JRIP propositional rule learner (RIPPER), and a 1‐R classifier. All these models can deal with the multiclass outcome and missing observations. Where possible, that is, concerning conditional inference trees, forests, and C5.0, observations of the unbalanced outcome classes PD, PSP, and MSA were weighted using Weights 1, 4, and 8. Parameter tuning, for example, concerning the number of boosting steps and the size of a tree, was performed by grid search in predefined values.

The outer cross‐validation loop was used for model selection, that is, to determine the ML method with the best average performance when fit to the combined data of the training and validation subsets and applied to the respective independent test subsets. Once the best ML method was determined, a respective and final model was fit to the entire dataset. Variable importance based on the Gini index was calculated for the final model (see also Figure S3).

The classification performance of a model was quantified by the multiclass area under the receiver operating characteristics curve (AUC) calculated as the mean AUC of the three binary classification tasks of one entity versus all other entities (Ferri, Hernández‐Orallo, and Modroiu 2009). To obtain unbiased estimates of discrimination (AUC) and model calibration, we used a stratified threefold nested cross‐validation. Thereby, class ratios were maintained in repeated splits of the data into training, validation, and test subsets. For an unbiased estimate of the performance that the final model (or, more generally, a best ML method) can achieve, the model selection was first repeated in the inner cross‐validation loops using the training subsets for model fitting and the validation subsets for performance evaluation. The models with the best average performance in the inner loops were then refitted to the combined training and validation subsets and applied to the respective independent test subsets of the outer loop. The average of the resulting AUC values served as an unbiased estimate of the final model's performance (see also Figure S4). Model calibration was assessed by comparing actual class frequencies versus the predicted probabilities produced by the inner loops’ best‐performing models for each clinical entity.

In addition, probability thresholds for binary classification tasks were modified to maximize Mathew's correlation coefficient (mcc). mcc is a measure for assessing the agreement of qualitative variables (i.e., predicted and clinical diagnosis). It takes a value of 0 if there is no association between the classifier results and clinical diagnosis and 1 for perfect classification. After applying the classification threshold thus obtained common classifier performance metrics were calculated for each binary classification task.

The significance of differences between disease entities among predicting variables was established using linear regression modeling with Huber–White corrections and Bonferroni correction for multiple testing for establishing pairwise differences in post hoc analysis using the R‐package “rms” (Harrell 2024). For a posteriori analysis of the influence of disease duration on the predicted probabilities, a linear regression model with disease duration, clinical diagnosis, and their interaction as regressors was estimated.

## 3 Results

### 3.1 Classification Performance—Discrimination

The primary purpose of our study was to separate different parkinsonian syndromes using imaging‐derived predictors alone and additional statements on the presence of clinical signs.

**FIGURE 1:**
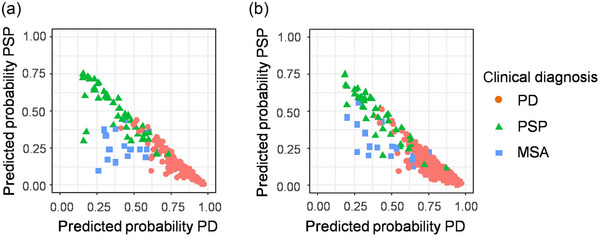
Individual probabilistic predictions. Each observation (i.e., each patient) is assigned three predicted probabilities, one belonging to each clinical entity (PD, PSP, and MSA). As probabilities add up to 1, it is sufficient to show the predicted probabilities, for example, PD and PSP. MSA observations are then expected to be located in the lower left corner. (a) Approach 1 combining clinical and imaging variables as predictors. (b) Approach 2 using only imaging variables as predictors.

Models based on both approaches assign each observation probability scores for belonging to a clinical entity (Figure 1). These probabilities were found to be independent of disease duration for both approaches (*p* = 0.77/0.45 for Approach 1/2) and the interaction of diagnosis and disease duration (*p* = 0.46/0.67 for Approach 1/2).

Considering Approach 1, combining clinical and imaging data, the best model in the outer loop of the nested cross‐validation was the AdaBoost.M1 tree with an average AUC of 0.948. An unbiased estimate of this best model's performance resulted in an AUC of 0.946.

In Approach 2, with the predictor space restricted to imaging variables, a Random Forest performed best in the outer cross‐validation loops (averaged AUC = 0.921). Its unbiased performance estimate was 0.921.

**FIGURE 2:**
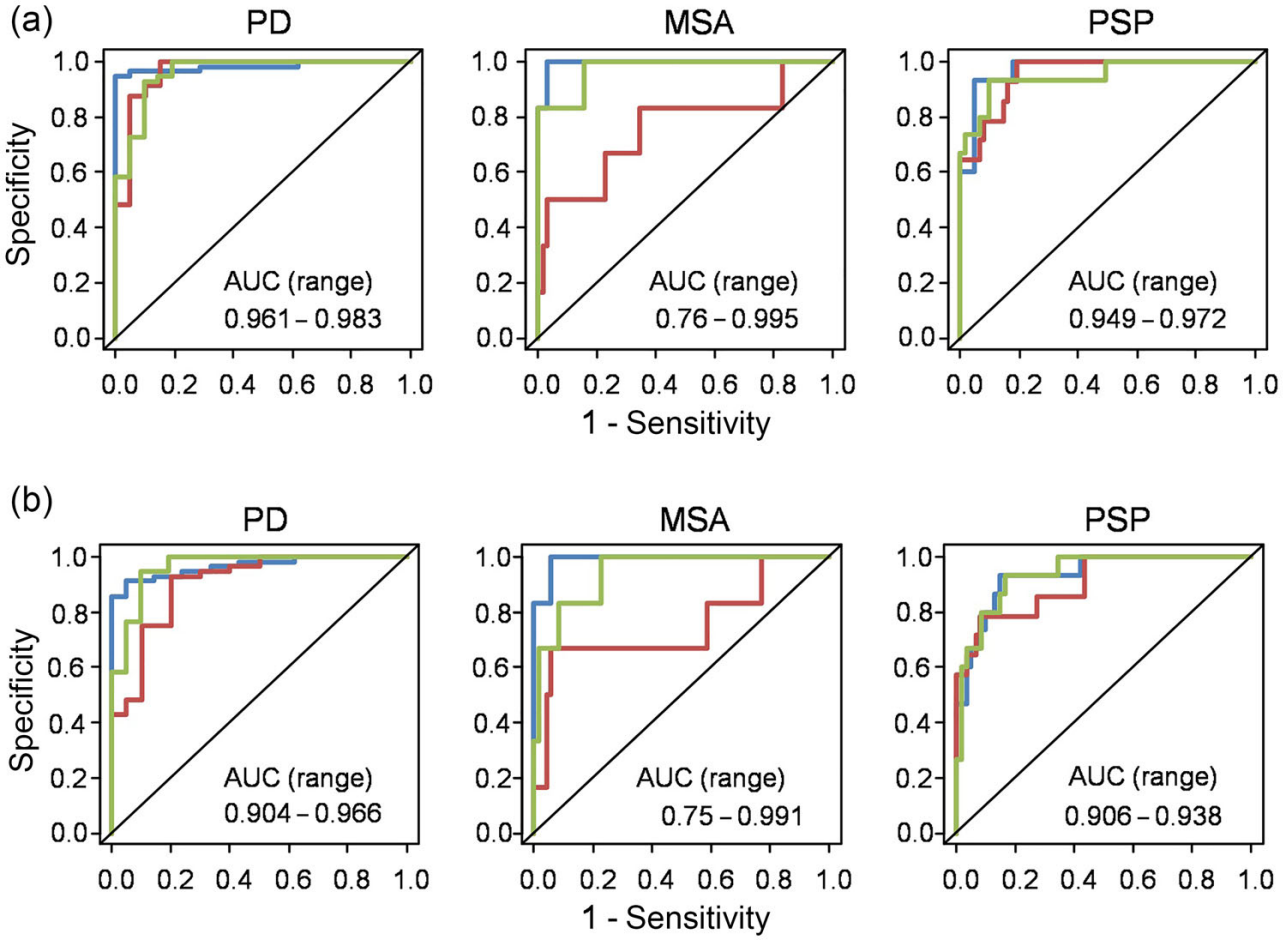
Receiver operator curves for the three binary classifications tasks. As each fold serves as a test fold once, three ROCs are shown, each representing the curve obtained by applying the best models of the inner cross‐validation loop to the corresponding test fold of the outer cross‐validation loop. Specificity and sensitivity at different probability thresholds were calculated within each test set to give rise to three ROC curves per binary classification task. (a) Approach 1 combining clinical and imaging variables as predictors. (b) Approach 2 using only imaging variables as predictors. Blue/orange/gray: AUCs calculated from test fold 1/2/3.

Regarding the binary classification tasks of individually classifying PD, PSP, and MSA each against the two other entities, the performance of the best‐performing models in the inner cross‐validation loop averaged over their respective test‐sets in the outer cross‐validation loop yielded AUCs of 0.969, 0.958, and 0.910 for Approach 1 and 0.945, 0.926, and 0.894 for Approach 2 (Figure 2).

### 3.2 Classification Performance—Prediction

Assessing model calibration, among observations with an intermediate assigned probability for MSA or PSP (0.33–0.66), the actual frequencies of the respective clinical diagnosis are 0.65/0.68 for PSP and 0.78/0.71 for MSA (Approach 1/2) (Figure S5). This hints toward a low sensitivity for aPS when using the default rule “allocate the diagnosis with the highest predicted probability.” Indeed, sensitivity/specificity were 1.00/0.56 and 0.98/0.50 for PD versus other, 0.59/1.00 and 0.55/0.96 for PSP versus other, and 0.33/0.99 and 0.17/1.00 for MSA versus other in Approaches 1 and 2. A better balance between sensitivity and specificity could be obtained by thresholds translating predicted probabilities to diagnoses that maximize mcc (Table 2). Please note that threshold adjustment is a posteriori analysis.

**TABLE 2:** Classifying performance using modified thresholds.

Task	AUC	sens	spec	ppv	npv	acc	bacc	mcc	*n*
(a) Approach 1: imaging and clinical variables									
PD vs. other	0.969	0.958	0.839	0.941	0.881	0.926	0.898	0.81	*n*_PD_ 167 *n*_other_ 62
PSP vs. other	0.958	0.682	0.968	0.833	0.927	0.913	0.825	0.703	*n*_PSP_ 44 *n*_other_ 185
MSA vs. other	0.920	0.611	0.986	0.786	0.967	0.956	0.798	0.670	*n*_MSA_ 18 *n*_other_ 211
(b) Approach 2: imaging variables only									
PD vs. other	0.945	0.946	0.758	0.913	0.893	0.895	0.852	0.728	*n*_PD_ 167 *n*_other_ 62
PSP vs. other	0.926	0.727	0.930	0.711	0.935	0.891	0.829	0.651	*n*_PSP_ 44 *n*_other_ 185
MSA vs. other	0.894	0.44	0.991	0.8	0.954	0.948	0.717	0.573	*n*_MSA_ 18 *n*_other_ 211

*Note*: The threshold maximizing mcc for each binary task was selected and the diagnosis were allocated if the predicted probability for the respective entity exceeded the selected threshold. Please note that threshold‐adjustment is a posteriori analysis.

Abbreviation: acc, accuracy; bacc, balanced accuracy; mcc, Mathew's correlation coefficient; npv, negative predictive value; ppv, positive predictive value; sens, sensitivity; spec, specificity.

### 3.3 Variable Importance

**FIGURE 3:**
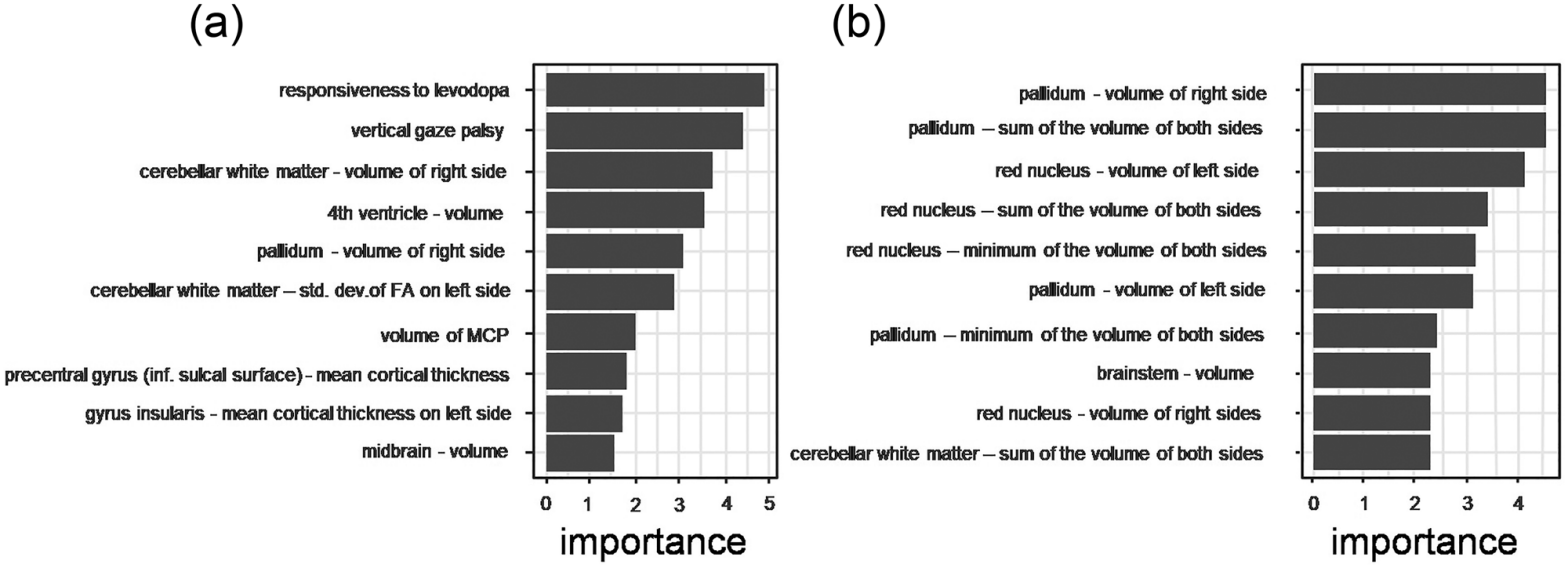
Variable importance: For the best performing model in both approaches Gini‐based importance measures for the predicting variables were calculated, and the importance score for the 10 most important predictor variables is depicted.

**FIGURE 4:**
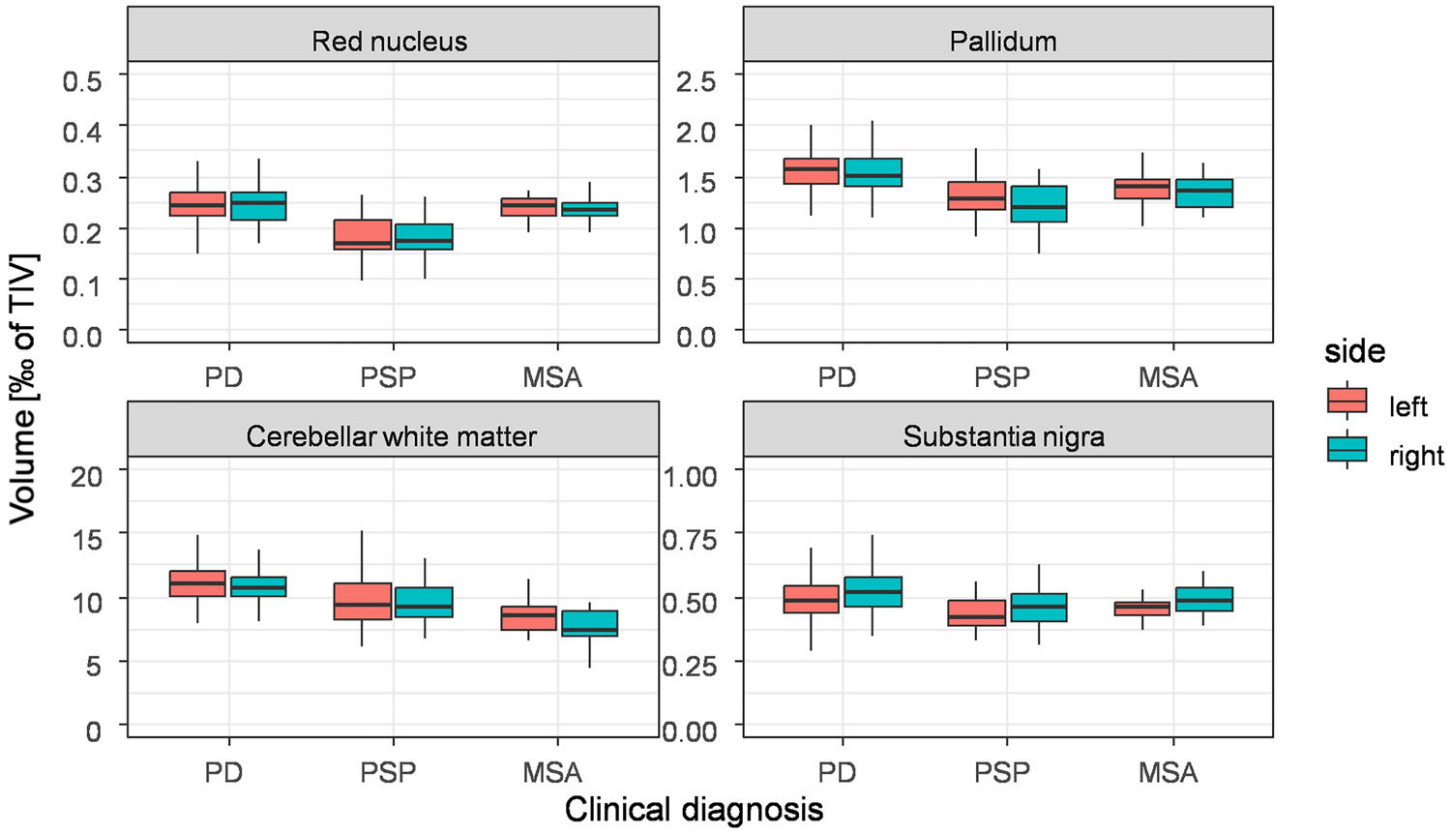
Volume of subcortical structures as segmented using MIST (red nucleus and substantia nigra) and FreeSurfer (pallidum and cerebellar white matter). For each structure and each side volumes were significantly different between disease entities (*p* < 0.001, linear regression with Huber–White correction). Pairwise testing, however (see text), was not significant for all pairwise tests conducted.

In the best model in Approach 1 vertical gaze palsy and responsiveness to levodopa were the variables most important for classification followed by volumes of subcortical structures like the volume of the right cerebellar white matter (Rank 3, 11.19/9.68/8.05 [‰ of TIV] PD/PSP/MSA, *p* < 0.001), the fourth ventricle (Rank 4, 1.77/2.33/2.80 [‰ of TIV], PD/PSP/MSA, *p* < 0.001), and the right pallidum (Rank 5 1.54/1.21/1.34 [‰ of TIV] PD/PSP/MSA, *p* < 0.001), as well as the standard deviation of FA in the left cerebellar white matter (Rank 6, 0.17/0.17/0.14 PD/PSP/MSA). In the best model in Approach 2, volumes related to the pallidum (right pallidum, the sum of both pallidal volumes, left pallidum, a minimum of pallidal volumes ranking 1, 2, 6, 7), and the red nucleus (left red nucleus, the sum of both red nuclei, minimum of the red nuclei, right red nucleus, ranking 3, 4, 5, 8) were among the most important variables for classification with volumes of the red nucleus (mean volumes of the left/right red‐nucleus 0.25/0.25, 0.18/0.18, 0.24/0.24 [‰ of TIV] in PD, PSP, MSA) reduced in PSP compared to PD and MSA (*p* < 0.001 each) but not in MSA compared to PD (*p* = 0.51/0.25 left/right) (Figures 3 and 4; Tables S2 and S3). Volumes of the pallidum (mean volume of the left/right pallidum 1.57/1.54, 1.31/1.21, 1.38/1.34 [‰ of TIV]) were reduced in PSP and MSA compared to PD (*p* < 0.001 each for both sides), but significantly reduced in PSP in comparison to MSA only for the right side (*p* = 0.33/0.04 [left/right]). In contrast, the volumes of the cerebellar white matter were smallest for MSA (mean volume of the left/right cerebellar white matter 11.42/11.19, 9.72/9.68, 8.75/8.05 [‰ of TIV]), significantly reduced in MSA and PSP compared to PD (*p* < 0.001 each for both sides) but significantly reduced in MSA compared to PSP only on the right side (*p* = 0.07/0.008 left/right). The volume of the substantia nigra was significantly reduced in PSP compared to PD (*p* < 0.001 on both sides) but not in MSA compared to PD (*p* = 0.48/0.98 left/right or PSP compared to MSA (*p* = 0.06/0.08 left).

## 4 Discussion

We compared several common ML algorithms for the simultaneous multinomial differentiation of the three major parkinsonian syndromes in a comparatively large single‐center collective of 229 patients. We were able to demonstrate excellent separation of entities and accurate confirmation of clinical diagnosis in binary classification tasks, which compare one entity versus all other entities. As a drawback however, like previous studies we found sensitivity for aPS in the multinomial case low.

### 4.1 Classifying Performance

Our benchmark study found that the best models are an AdaBoost.M1 tree when imaging and clinical information are used and a random forest when only imaging information is used. Overall, the approaches provided ‘excellent discrimination’ (Hosmer and Lemeshow 2000) of the entities (expected multiclass [PD, PSP or MSA vs. other] AUCs ≥ 0.92, averaged binary [one entity vs. the other two] AUCs ≥ 0.89). Comparing these results with the existing literature is only possible to a limited extent due to methodological differences. The three previous large‐scale imaging studies investigated the differential diagnosis of the three major parkinsonian syndromes via binary comparison of each parkinsonian syndrome by estimating separate models for this specific purpose. In contrast, we derived the predicted probabilities for this classification from a multinomial model. We chose this approach because it reflects clinical practice in which the neurologist must choose between more than one diagnosis.

An immediate comparison with the previous literature is possible for the comparison of the binary “Parkinson's versus other.” Here, our classification results (0.97/0.95 with/without clinical variables) with a sensitivity/specificity combination of 0.96/0.84 (with) and 0.95/0.76 (without clinical variables) after threshold adjustment for AUC are in the same range as the previous binomial classification studies using volumetry, DTI or volumetry‐DTI combinations (AUCs: n.a./0.91/0.96; sensitivity/specificity combinations 0.82/0.87, 0.90/0.79, 0.90/0.90 [Archer et al. 2019; Chougar et al. 2021; Huppertz et al. 2016]). To provide meaningful sensitivity and specificity figures, we had to adjust the classification threshold in the a posteriori analysis, which carries some risk of positive bias. As we consider the discriminatory power measured by AUC to be the main outcome and we chose to maximize the mcc rather than, for example, the balanced accuracy (the sum of specificity and sensitivity), we consider this risk to be acceptable.

As mentioned above, multinomial classification (assignment to either PD, PSP, or MSA), as applied in our work, is a more practical but more difficult task than the binary approach. While one classification study reported multinomial classification accuracy comparable to that in the binary case (Huppertz et al. 2016), this has not been reproduced in the two more recent studies on this topic. Chougar et al. (2021) reported low sensitivities for PSP and MSA (0.73 and 0.69) in a multinomial model. If one calculates corresponding overall sensitivities from the sensitivity and specificity values published by Archer and colleagues of their two‐stage binary classification approach (PD vs. aPS, followed by MSA vs. PSP) that enable comparison with our data, one would arrive at an overall sensitivity for MSA or PSP of 0.79 and 0.69, respectively (Archer et al. 2019).

Several limitations are potentially relevant here. The higher‐class imbalance in our cohort may have resulted in lower sensitivity, especially for MSA. As PD cases far outnumber PSP and MSA cases in the general population (prevalence of PSP/MSA 5–10/100,000 vs. PD 108–257/100,000 [Levin et al. 2016; von Campenhausen et al. 2005]), class imbalance is an inherent issue in representative datasets of parkinsonian syndromes, affecting almost all studies. Class imbalance challenges ML methods, which tend to assign higher predicted probabilities to more prevalent classes (Kuhn 2013, 419 onwards). Our sample distribution, though more balanced than the general population, reflects the frequency distribution in a specialist outpatient clinic. Besides disease frequencies, other characteristics of the patient collective may affect classification study results. The lack of available standardized assessments (UPDRS‐III, PSPRS, UMSARS) (Goetz et al. 2008; Golbe and Ohman‐Strickland 2007; Wenning et al. 2004) could limit comparability with other studies. However, such assessments also have limitations, such as ceiling effects over time and reliability issues due to therapeutic interventions (Evers et al. 2019; Palma et al. 2021). The disease duration in our study, which could serve as a suitable proxy for disease severity, was comparable to other classification studies.

### 4.2 The Role of Clinical Insights

It was of note that the presence of clinical variables improved the separability of parkinsonian syndromes as indicated by a higher AUC (0.95/0.92 with/without clinical variables), but did not have a sweeping impact, although partial circularity is inherent to this approach considering the composition of clinical diagnostic criteria for MSA and PSP. In particular, the addition of clinical information in PSP only led to a slight increase in sensitivity compared to the imaging‐only approach. For MSA, there was a clearer advantage compared to sensitivity based on imaging markers only when clinical information was considered. The former was especially interesting as following responsiveness to levodopa, the second most important clinical variable was deemed vertical gaze palsy. The qualitative character of the clinical information may have played a role here, yet we opted for this assessment as we considered robust (reproducible) descriptive graduation of some symptoms difficult, especially when being assessed by non‐movement‐disorders specialists—which would be the initial situation for the majority of these patients in routine practice.

### 4.3 Imaging Variables: The Importance of the Red Nucleus

Regarding neuroimaging variables, volumetric measures were, in general, overrepresented among the most discriminatory variables compared to DTI metrics. While the low number of gradient directions (owed to our effort to minimize scanning time within the multimodal MRI protocol) could have been a relevant contributing factor, the overall directionality of this observation is in line with a multimodal study that included high‐quality diffusion imaging (Chougar et al. 2021). DTI has been used for the differentiation of PSP and PD (Potrusil et al. 2020; Seki et al. 2018; Talai et al. 2018) as well as MSA and PD (Beliveau et al. 2021; Krismer et al. 2021) as well as to differentiate all three syndromes (Archer et al. 2019). As DTI is technically more challenging regarding patient eligibility, acquisition, processing, and analysis, comparative evaluations of DTI‐ and volumetry‐based assessments in future studies should clarify if pure volumetric studies may suffice for classification.

Subcortical structure volumes were the most crucial among individual imaging variables. While the volume of the substantia nigra is differentially affected between diseases and reduced in PSP compared to PD, its volume—in line with findings in SPECT and SWI‐Imaging (Bae et al. 2016; Booth et al. 2015)—was not as informative for differentiating between the three clinical entities. Moreover, volumetry based on standard images might not be able to fully capture pathologic changes in the substantia nigra, as evidenced by the widespread use of more sophisticated iron‐sensitive imaging for assessing this structure (Bae et al. 2021).

The observation of the reduced volume of the red nucleus in PSP in our imaging‐only approach was intriguing. Mesencephalic atrophy is a well‐known PSP indicator (Mangesius et al. 2018; Moller et al. 2017; Mueller et al. 2018; Sjöström et al. 2020). Our segmentation of mesencephalic structures using a multimodal approach with automated volumetry could indicate that some mesencephalic regions may show predominant atrophy. Previous studies suggested alterations in the dentato‐rubral tract using DTI, and iron and tau deposits are observed in the red nucleus in PSP patients (Dickson 2012; Koga, Zhou, and Dickson 2021; Kouri et al. 2021; Li et al. 2021; Mazzucchi et al. 2019; Potrusil et al. 2020; Seki et al. 2018; Sjöström et al. 2017; Surova et al. 2015). Our findings advocate for including the red nucleus in future imaging analyses of parkinsonian syndromes.

### 4.4 ML Classifications in Clinical Practice Amid Diagnostic Uncertainty

For clinical practice, a model that can distinguish at least three common parkinsonian syndromes is desirable. We have shown the separability of these entities through a high multiclass AUC. However, diagnoses predicted by a classifier trained on clinically‐defined diagnoses face the same uncertainties as the clinical diagnoses themselves, which needs addressing to avoid a false sense of diagnostic certainty. Therefore, we suggest reporting not only predicted class labels but also the predicted probabilities for a patient's diagnosis. This approach allows for assessing the confidence in a predicted diagnosis. Predicted probabilities can be interpreted both numerically and visually. Numerically, patients exceeding a certain probability threshold could, for example, be enrolled in clinical trials. Visually, results can be checked to see which cluster a patient fits into, as shown in Figure 1. This visualization helps identify patients who do not fit their expected cluster and may need closer monitoring during follow‐up.

## 5 Conclusion

In summary, we evaluated two neuroimaging‐based approaches to support the differential diagnosis of parkinsonian syndromes. While the reduced sensitivity remains a problem for potential early diagnosis in purely neuroimaging‐based approaches, we also saw that including basic clinical information cannot fundamentally overcome this. It remains to be seen whether an improvement can be achieved in the future through extended imaging sequences or the addition of other objective markers. Nevertheless, imaging diagnostics can be a valuable support clinical routine. To this end, we propose a probabilistic view of the diagnosis with corresponding visualization of the classifier results, which can provide guidance in clinical practice.

## Author Contributions

**Tobias Meindl**: conceptualization, methodology, data curation, investigation, writing‐original draft, formal analysis. **Alexander Hapfelmeier**: conceptualization, formal analysis, writing‐original draft, methodology. **Tobias Mantel**: writing‐review and editing, investigation. **Angela Jochim**: investigation, writing‐review and editing. **Jonas Deppe**: writing‐review and editing, investigation. **Silke Zwirner**: investigation, writing‐review and editing. **Jan S. Kirschke**: resources. **Yong Li**: writing‐review and editing, methodology. **Bernhard Haslinger**: conceptualization, writing‐review and editing, resources, supervision, investigation.

## Ethics Statement

The study has been approved by the local ethics review board (Ethikkommission der Technischen Universität München). Patients whose data were not only acquired retrospectively gave their written informed consent before entering the prospective phase of the study. Informed consent was waived from patients whose data was used only retrospectively.

## Conflicts of Interest

The authors declare no conflicts of interest.

### Peer Review

The peer review history for this article is available at https://publons.com/publon/10.1002/brb3.70274.

## Supporting information



Supporting Information

## Data Availability

The data supporting the findings of this study are available on request from the corresponding author. The data are not publicly available due to privacy or ethical restrictions.
