# Supplementary material for: Assisted Parkinsonism Diagnosis Using Multimodal MRI—The Role of Clinical Insights
Source: Brain Behav. 2025 Jan 19;15(1):e70274. doi: 10.1002/brb3.70274 (PMC11743991; doi:10.1002/brb3.70274)
Supplement: Supplementary file 1 — Supporting Information [file BRB3-15-e70274-s001.docx]

**Supplementary Information**

**To**

**Assisted parkinsonism diagnosis using multimodal MRI - the role of clinical insights**

Tobias Meindl^a1^, Alexander Hapfelmeier^b,d1^, Tobias Mantel^a^, Angela Jochim^a^, Jonas Deppe^a^; Silke Zwirner^a^, Jan S. Kirschke^c^, Yong Li^a^, Bernhard Haslinger^a^*

1. **Supplementary Methods:**

**1.1. Image Acquisition and Processing**

Sequences used during data acquisition are outlined below in table s1.

| **Supplementary table s1**: **Details on sequence parameters**: | | | | | | | | | |
| --- | --- | --- | --- | --- | --- | --- | --- | --- | --- |
|  | N | Direction of acquisition | Voxel-Size [mm] | TE [ms] | TR [ms] | T IR [ms] | Gradient directions | B  [s/mm^2^] | Duration [s] |
| T1 Sequences |  |  |  |  |  |  |  |  |  |
| 3 (most current) | 89 | Sagittal | 1x1x1 | 4 | 9 | 1000 |  |  | 145 |
| 2 | 41 | Sagittal | 1x1x1 | 4 | 9 | 1000 |  |  | 365 |
| 1 | 99 | Sagittal | 1x1x1 | 3,8 | 7,7 | 1200 |  |  | 279 |
| FLAIR Sequences |  |  |  |  |  |  |  |  |  |
| 2 (most current) | 89 | Sagittal | 1x1x1 | 290 | 4800 | 1650 |  |  | 235 |
| 1 | 140 | Sagittal | 1x1x1 | 325 | 4800 | 1650 |  |  | 373 |
| SWI | 82 | Transversal | .75x.75x1.5 | 6 echoes, spacing 6ms | 38 |  |  |  | 199 |
| DTI | 188 | Transversal | 2x2x2 | 55 | 7529 |  | 6 | 1000 | 144 |
| T1-Sequence 3 and FLAIR sequence 2 as well as the SWI images were acquired on scanner 2. Here, shorter scan durations were enabled using compressed sensing. The remaining T1 and FLAIR sequences were acquired on scanner 1, the DTI sequence-parameters were identical on both scanners. | | | | | | | | | |

Software used: Anatomical scans were processed in CAT12 (r1152, http://www.neuro.uni-jena.de/cat/), FreeSurfer (v. 6.0, https://surfer.nmr.mgh.harvard.edu/) and MIST (https://fsl.fmrib.ox.ac.uk/fsl/fslwiki/MIST). dMRI were preprocessed using ExploreDTI (v.4.8.6, www.exploredti.com) and SPM (r7219, <https://www.fil.ion.ucl.ac.uk/spm/>).

**1.2. Anatomical-images – Quality control, parcellationd**

The processing stream for the analysis of T1, FLAIR and SWI images for volumetry was divided into two substreams.

First, CAT12 was employed for analysis of T1-weighted images: After skull-stripping, segmentation, and DARTEL-normalization to MNI-space the measures derived were a quantitative measure of noise to contrast ratio (Dahnke et al., unpublished work, personal correspondence, http://www.neuro.uni-jena.de/cat12-html/cat_methods_QA.html), and estimates of the total intracranial volume (TIV), total white and gray matter volume as well as the volume of the middle cerebellar peduncle (MCP) as defined by the JHU white matter atlas [1] from T1-images (Supplementary Figure s1, Analysis pathway Ia).

Skull-stripped T1-images generated with CAT12 together with FLAIR-images for better delineation of the pial surface were fed into FreeSurfer (Supplementary Figure s1, Analysis pathway Ia). The cortical ROIs analyzed were cortical regions defined by the Destrieux-atlas [2]. FreeSurfers subcortical stream [3] led to ROI-masks and ROI volumes for cerebellar gray and white matter, amygdala, Ncl. accumbens, thalamus, caudate, putamen, pallidum, hippocampus, lateral, 3^rd^, 4^th^ ventricle, total and hemispheric gray and white matter volume, subcortical gray matter volume. Finally, FreeSurfer’s brainstem stream [4] gave rise to ROI-masks and volumes of the following structures: midbrain, pons, medulla oblongata and superior cerebellar peduncle.

In a second stream, for parcellation of substantia nigra, red nucleus and subthalamic nucleus multimodal parcellation was performed using MIST. For this purpose, after coregistration of FLAIR, T2 and SWI to T1 via SPM and skull-stripping with BET a model was first trained using 120 scans. This model was then applied to T1 and FLAIR-images of all subjects (Supplementary Figure s1, Analysis pathway Ib).


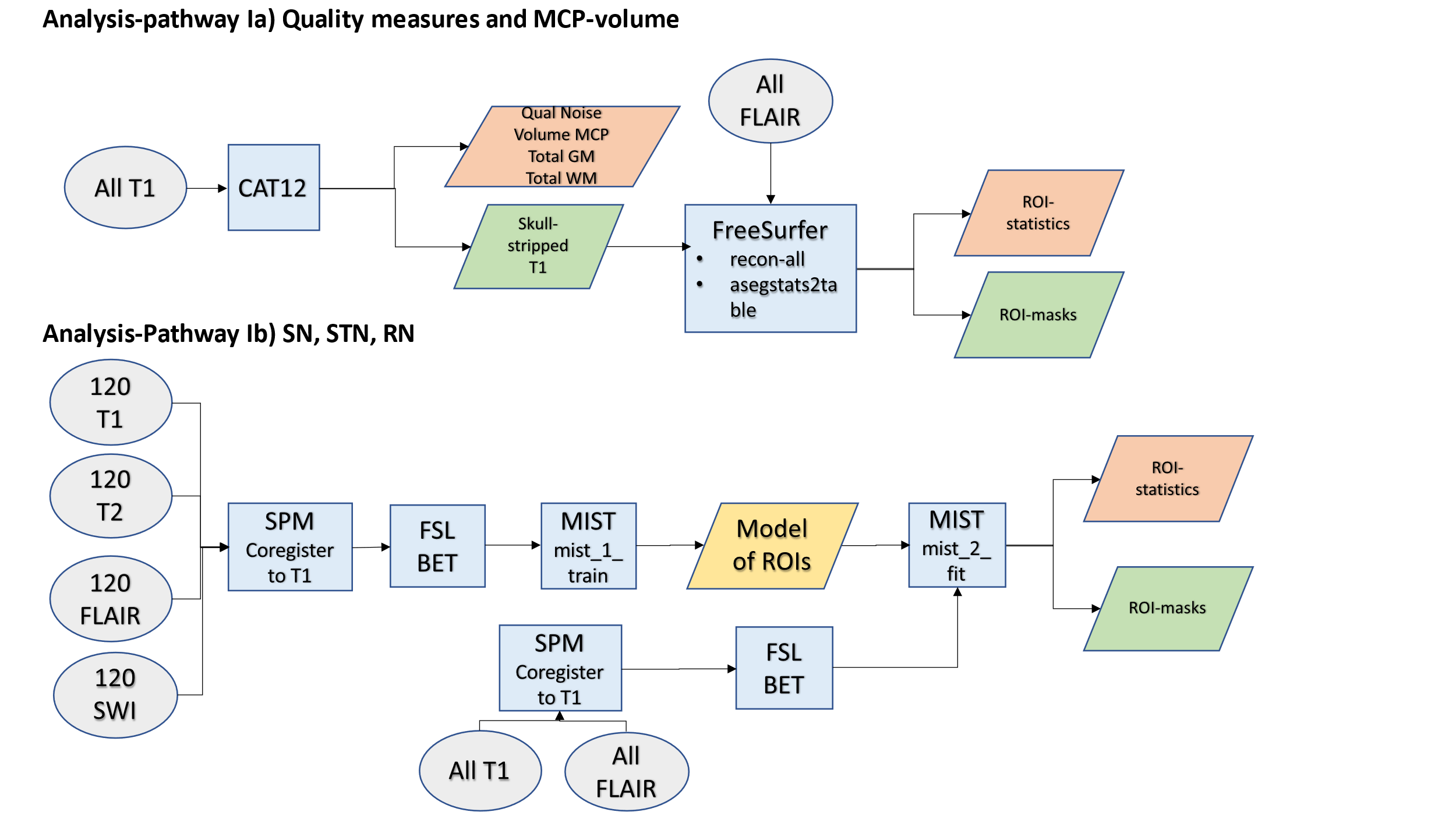


**Supplementary figure s1:** Processing of anatomical images: The goal of image processing is to obtain ROI-masks for further processing and ROI-based statistics. CAT12: Computational Anatomy Toolbox, MIST: Multimodal Image Segmentation Tool, BET: Brain Extraction Toolbox, SPM: Statistic Parametric Mapping, MCP: middle cerebellar peduncle, GM: gray matter, WM: white matter, SN: substantia nigra, STN subthalamic nucleus, RN: red nucleus. Grey ovals: original images. Blue rectangulars: processing steps. Red trapezoids: numeric results of image processing for subsequent machine-learning analysis. Green trapezoids: Images/ROI-masks resulting from processing steps. Yellow trapezoids intermediary data. For FreeSurfer, MIST and SPM processing steps the functions used are stated.

- 1. **DTI-Analysis**

Gibbs-Ringing-correction, motion/distortion-correction and Eddy-current-correction were performed using ExploreDTI which was also used to generate FA- and MD-maps. Afterwards, these maps were either coregistered to a T1 preprocessed with FreeSurfer to obtain ROI-based statistics for ROIs parcellated with FreeSurfer, or coregistered to native T1 to obtain statistics for the ROIs parcellated with MIST. For the latter, FreeSurfer functionality was employed to obtain the statistics (Supplementary Figure s2).

**
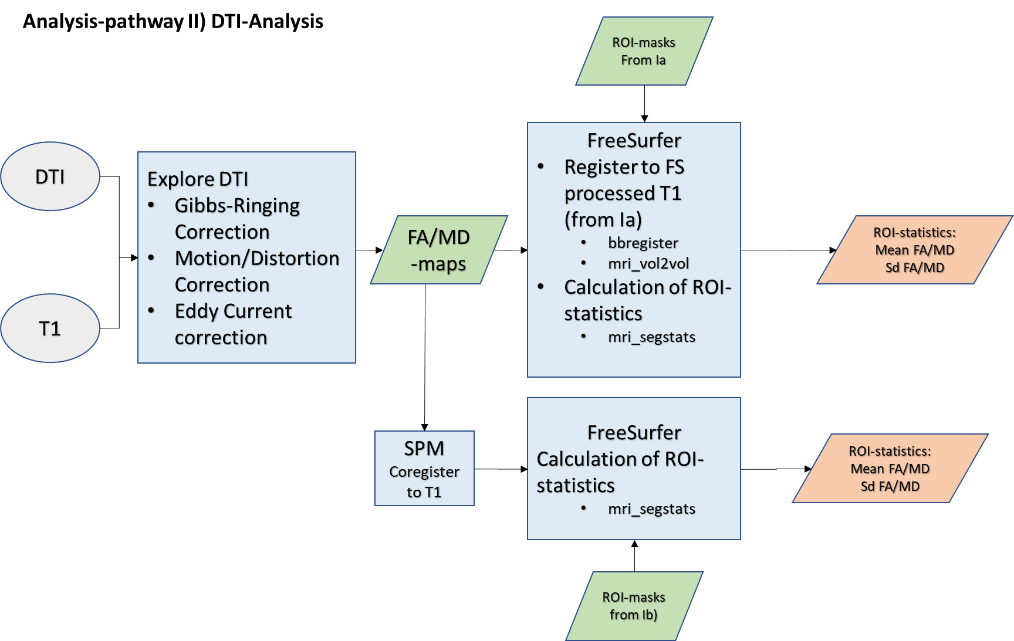
**

**Supplementary figure 2:** Processing of DTI-Images. The ultimate goal of the processing streams are ROI based statistics of the ROIs parcellated in Analysis pathway I. For FreeSurfer- and SPM-processing steps the functions used are stated.

1. **Supplementary manuscript tables and figures**

**Machine Learning Classification:**

**
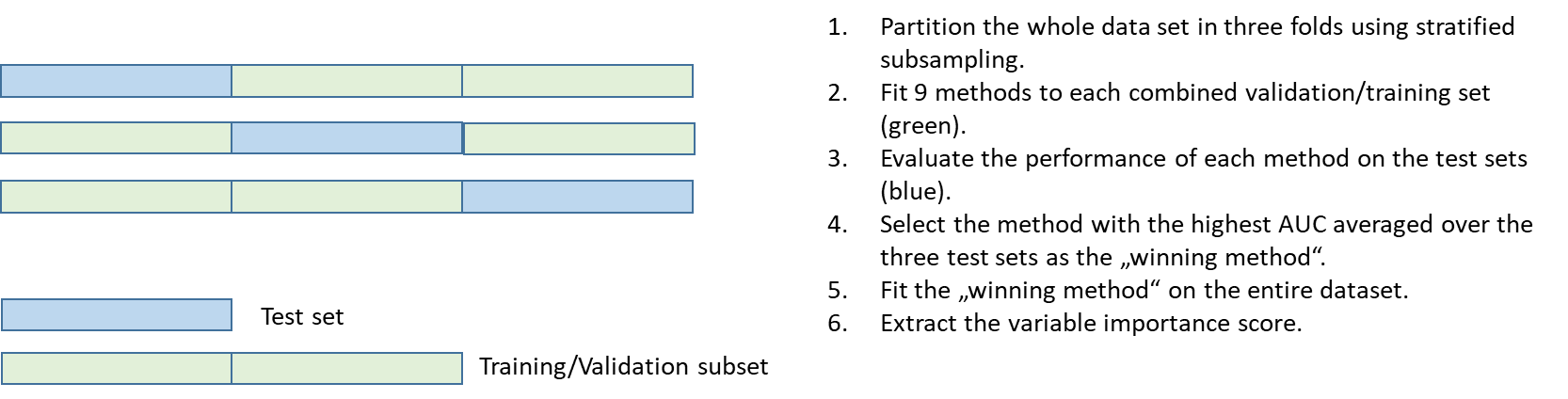
**

**Supplementary figure 3**: **Selection of the best model and extraction of the variable-importance scores**


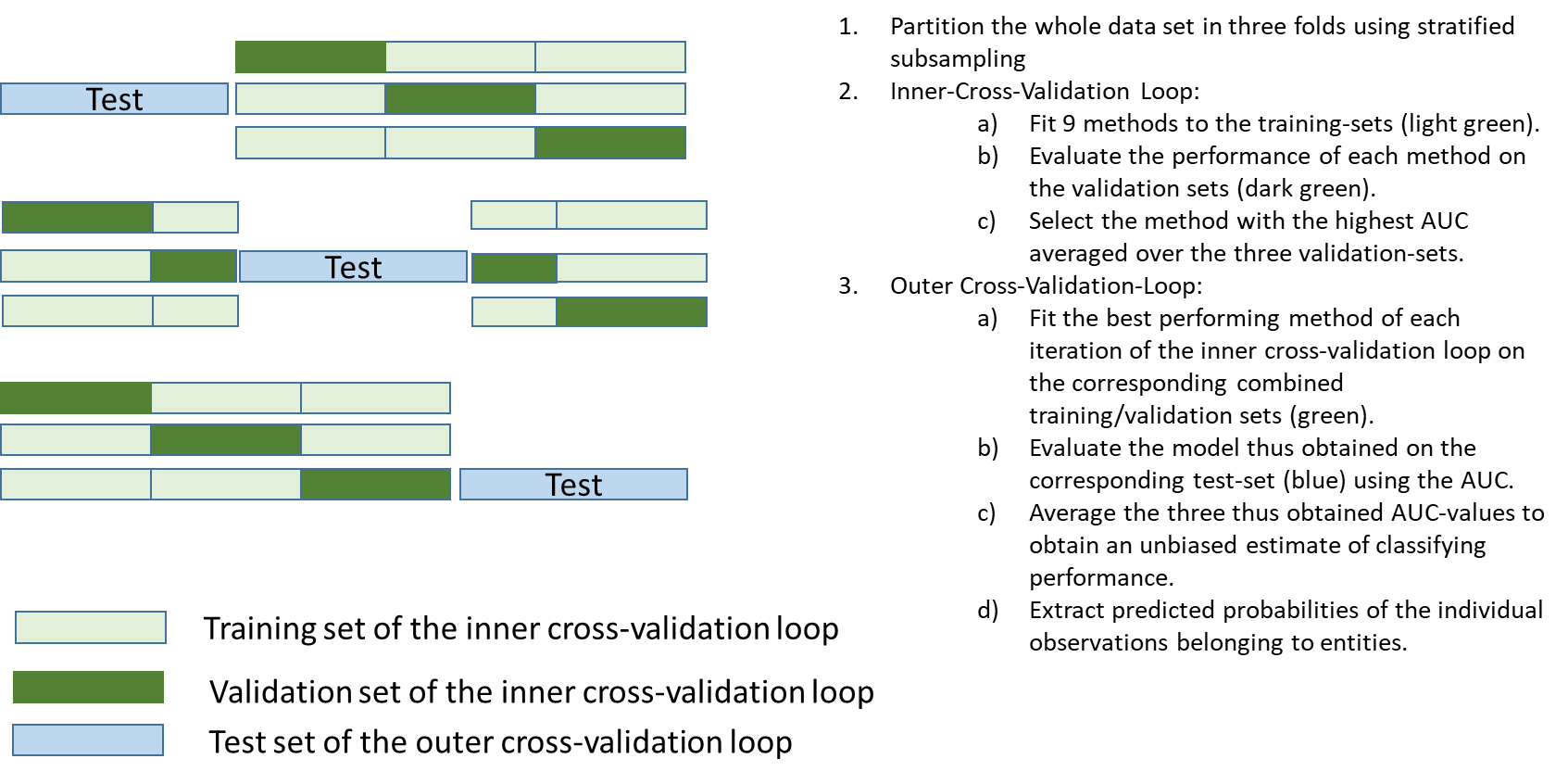


**Supplementary figure s4**: **Estimation of unbiased classifying performance and calculation of individual predictions.**

**A)**

**B)**

**Supplementary figure s5: Model Calibration:** The machine-learning procedure assigns three probabilities to each observation - one for belonging to each clinical diagnosis. For illustration, observations of each clinical diagnosis are grouped into observations with low, intermediate and high predicted probabilities for their clinical diagnosis. Then, for each group and each clinical entity the mean predicted probability is calculated and plotted vs. the actual frequency of the respective entity in this group. A) Approach 1using clinical and imaging predictor variables B) Approach 2 with imaging variables only.

| **Supplementary Table s2: Variable Importance – Approach 1: Using Imaging and clinical variables** | | | | | | |
| --- | --- | --- | --- | --- | --- | --- |
| **rank** | **Variable** | **importance** | **IPS** | **PSP** | **MSA** | **p** |
| 1 | **responsiveness to levodopa** | 4.90 |  |  |  | < 0.001 |
|  | Positive |  | 126 | 7 | 7 |  |
|  | possibly positive |  | 7 | 9 | 3 |  |
|  | Negative |  | 2 | 15 | 4 |  |
|  | Missing |  | 32 | 13 | 4 |  |
| 2 | **vertical gaze palsy** | 4.46 |  |  |  | < 0.001 |
|  | not present |  | 148 | 9 | 14 |  |
|  | Present |  | 14 | 34 | 3 |  |
|  | Missing |  | 5 | 1 | 1 |  |
| 3 | **volume right cerebellar white matter** | 3.76 | 11.19 ± 2.28 | 9.68 ± 1.94 | 8.05 ± 2.11 | < 0.001 |
| 4 | **volume 4th ventricle** | 3.54 | 1.77 ± 0.50 | 2.33 ± 0.60 | 2.80 ± 0.85 | < 0.001 |
| 5 | **volume right pallidum** | 3.10 | 1.54 ± 0.19 | 1.21 ± 0.21 | 1.34 ± 0.21 | < 0.001 |
| 6 | **standard deviation of FA left cerebellar white matter** mean SD | 2.90 | 0.17 ± 0.01 | 0.17 ± 0.01 | 0.14 ± 0.02 | < 0.001 |
|  | Missing |  | 33 | 5 | 3 |  |
| 7 | **volume MCP** | 1.99 | 9.63 ± 1.03 | 8.69 ± 1.12 | 7.56 ± 1.54 | < 0.001 |
| 8 | **mean cortical thickness left precentral gyrus (inferior sulcal surface)** | 1.89 | 2.54 ± 0.32 | 2.41 ± 0.15 | 2.55 ± 0.15 | 0.024 |
| 9 | **mean cortical thickness left gyrus insularis (short)** | 1.71 | 3.45 ± 0.51 | 3.52 ± 0.28 | 3.60 ± 0.31 | < 0.001 |
| 10 | **volume midbrain** | 1.55 | 5.42 ± 0.40 | 4.80 ± 0.44 | 4.99 ± 0.56 | < 0.001 |
| 11 | **rest tremor** | 1.47 |  |  |  | < 0.001 |
|  | Present |  | 87 | 3 | 2 |  |
|  | not present |  | 78 | 37 | 16 |  |
|  | Missing |  | 2 | 4 | 0 |  |
| 12 | **mean FA in left gyrus rectus** | 1.32 | 0.25 ± 0.05 (33) | 0.24 ± 0.05 (5) | 0.30 ± 0.04 (3) | 0.0001 |
| 13 | **minimum volume of pallidum** | 1.30 | 1.49 ± 0.19 | 1.19 ± 0.19 | 1.31 ± 0.22 | < 0.001 |
| 14 | **minimum volume of volume of vertical column of anterior fissure** | 1.28 | 0.29 ± 0.10 | 0.23 ± 0.06 | 0.30 ± 0.06 | 0.0004 |
| 15 | **volume of right putamen** | 1.26 | 3.65 ± 0.77 | 3.33 ± 0.41 | 3.14 ± 0.64 | 0.0014 |
| 16 | **standard deviation of FA in right cerebellar white matter** | 1.24 | 0.17 ± 0.01 (33) | 0.17 ± 0.01 (5) | 0.14 ± 0.02 (3) | < 0.001 |
|  |  |  |  |  |  |  |
| 17 | **volume pons** | 1.22 | 13.15 ± 1.23 | 11.90 ± 1.19 | 10.52 ± 2.19 | < 0.001 |
| 18 | **minimum volume of red nucleus** | 1.10 | 0.24 ± 0.04 (6) | 0.17 ± 0.04 (2) | 0.23 ± 0.03 (1) | < 0.001 |
| 19 | **volume SCP** | 1.07 | 0.24 ± 0.04 | 0.20 ± 0.04 | 0.18 ± 0.03 | < 0.001 |
| 20 | **minimum volume of bilateral cerebellar white matter** | 1.06 | 10.96 ± 2.14 | 9.37 ± 1.92 | 7.82 ± 1.91 | < 0.001 |
| 21 | **sum of volume of pallidum** | 1.03 | 3.11 ± 0.36 | 2.52 ± 0.39 | 2.72 ± 0.40 | < 0.001 |
| 22 | **age at MRI** | 1.03 | 65.42 ± 10.45 | 70.59 ± 7.60 | 58.94 ± 7.18 | < 0.001 |
| 23 | **volume total white matter** | 1.02 | 440 ± 30 | 420 ± 30 | 460 ± 20 | < 0.001 |
| 24 | **volume left red nucleus** | 0.97 | 0.25 ± 0.04 (6) | 0.18 ± 0.04 (2) | 0.24 ± 0.03 (1) | < 0.001 |
| 25 | **volume left pallidum** | 0.96 | 1.57 ± 0.20 | 1.31 ± 0.21 | 1.38 ± 0.21 | < 0.001 |
| 26 | **standard deviation of cortical thickness of lateral orbital sulcus** | 0.91 | 0.40 ± 0.13 | 0.37 ± 0.09 | 0.40 ± 0.13 | 0.21294 |
| 27 | **volume right red nucleus** | 0.90 | 0.25 ± 0.08 (6) | 0.18 ± 0.04 (2) | 0.24 ± 0.04 (1) | < 0.001 |
| 28 | **FS_WM_volume_wm_rh_caudalanteriorcingulate** | 0.86 | 2.29 ± 0.65 | 2.08 ± 0.26 | 2.40 ± 0.33 | 0.05582 |
| 29 | **volume right lateral ventricle** | 0.83 | 13.92 ± 7.42 | 19.54 ± 8.70 | 11.37 ± 5.96 | < 0.001 |
| 30 | **volume white matter in right precentral gyrus** | 0.83 | 13.31 ± 1.85 | 11.71 ± 1.56 | 13.45 ± 1.46 | < 0.001 |

**Supplementary table s2:** **Approach 1 combining clinical and imaging derived variables – Variable Importance** of the 30 most discriminative variables. For numeric variables, the significance of the diagnosis being discriminatory is assessed using linear (numeric variables) or logistic (categorial variables) regression. Volumes are presented as volume of the ROI as fraction of the total intracranial volume [‰]. Cortical thickness is presented in mm. Values are presented as mean ± sd If missing values occur in a variable, their number is stated in parenthesis.

| **Supplementary Table s3: Variable Importance – Approach 2 using imaging variables only** | | | | | | |
| --- | --- | --- | --- | --- | --- | --- |
| **rank** | **Variable** | **importance** | **IPS** | **PSP** | **MSA** | **p** |
| 1 | **volume right pallidum** | 0.00447619 | 1.54 ± 0.19 | 1.21 ± 0.21 | 1.34 ± 0.21 | p < 0.001 |
| 2 | **sum volume of bilateral pallidum** | 0.00447619 | 3.11 ± 0.36 | 2.52 ± 0.39 | 2.72 ± 0.40 | p < 0.001 |
| 3 | **volume left red nucleus** | 0.00404762 | 0.25 ± 0.04 (6) | 0.18 ± 0.04 (2) | 0.24 ± 0.03 (1) | p < 0.001 |
| 4 | **sum volume of bilateral red nucleus** | 0.00335714 | 0.50 ± 0.11 | 0.37 ± 0.08 | 0.48 ± 0.07 | p < 0.001 |
|  | Missing |  | 6 | 2 | 1 (5.56%) |  |
| 5 | **minimum volume of red nucleus** | 0.00311905 | 0.24 ± 0.04 (6) | 0.17 ± 0.04 (2) | 0.23 ± 0.03 (1) | p < 0.001 |
| 6 | **volume left pallidum** | 0.00307143 | 1.57 ± 0.20 | 1.31 ± 0.21 | 1.38 ± 0.21 | p < 0.001 |
| 7 | **minimum volume of bilateral pallidum** | 0.00240476 | 1.49 ± 0.19 | 1.19 ± 0.19 | 1.31 ± 0.22 | p < 0.001 |
| 8 | **volume right red nucleus** | 0.00226191 | 0.25 ± 0.08 (6) | 0.18 ± 0.04 (2) | 0.24 ± 0.04 (1) | p < 0.001 |
| 9 | **volume brainstem** | 0.00226191 | 18.83 ± 1.62 | 16.55 ± 1.64 | 15.98 ± 2.48 | p < 0.001 |
| 10 | **sum of volume bilateral cerebellar white matter** | 0.00226191 | 22.61 ± 4.49 | 19.40 ± 3.82 | 16.81 ± 3.59 | p < 0.001 |
| 11 | **volume left thalamus** | 0.00219048 | 6.03 ± 0.84 | 5.27 ± 0.53 | 6.03 ± 0.64 | p < 0.001 |
| 12 | **volume right cerebellar white matter** | 0.00219048 | 11.19 ± 2.28 | 9.68 ± 1.94 | 8.05 ± 2.11 | p < 0.001 |
| 13 | **volume 4th ventricle** | 0.00188095 | 1.77 ± 0.50 | 2.33 ± 0.60 | 2.80 ± 0.85 | p < 0.001 |
| 14 | **volume midbrain** | 0.00176191 | 5.42 ± 0.40 | 4.80 ± 0.44 | 4.99 ± 0.56 | p < 0.001 |
| 15 | **volume whole brainstem** | 0.00176191 | 23.03 ± 1.85 | 20.85 ± 1.78 | 19.54 ± 2.91 | p < 0.001 |
| 16 | **minimum of bilateral cerebellar white matter** | 0.00176191 | 10.96 ± 2.14 | 9.37 ± 1.92 | 7.82 ± 1.91 | p < 0.001 |
| 17 | **volume left cerebellar white matter** | 0.00171429 | 11.42 ± 2.34 | 9.72 ± 1.98 | 8.75 ± 1.67 | p < 0.001 |
| 18 | **sum volume of bilateral ventral diencephalon** | 0.00164286 | 6.50 ± 0.62 | 5.60 ± 0.68 | 6.26 ± 0.60 | p < 0.001 |
| 19 | **volume MCP** | 0.00161905 | 9.63 ± 1.03 | 8.69 ± 1.12 | 7.56 ± 1.54 | p < 0.001 |
| 20 | **volume pons** | 0.00147619 | 13.15 ± 1.23 | 11.90 ± 1.19 | 10.52 ± 2.19 | p < 0.001 |
| 21 | **volume SCP** | 0.00140476 | 0.24 ± 0.04 | 0.20 ± 0.04 | 0.18 ± 0.03 | p < 0.001 |
| 22 | **volume subcortical gray matter** | 0.00130952 | 45.00 ± 4.30 | 41.12 ± 2.75 | 43.25 ± 3.64 | p < 0.001 |
| 23 | **mean MD in left thalamus** | 0.00114286 | 0.00 ± 0.00 (33) | 0.00 ± 0.00 (5) | 0.00 ± 0.00 (3) | p < 0.001 |
| 24 | **minimum of bilateral volume of the ventral diencephalon** | 0.00111905 | 3.18 ± 0.33 | 2.71 ± 0.35 | 3.05 ± 0.32 | p < 0.001 |
| 25 | **sum of volume of bilateral thalamus** | 0.00111905 | 11.55 ± 1.38 | 10.27 ± 0.88 | 11.59 ± 1.11 | p < 0.001 |
| 26 | **volume right ventral dicencephalon** | 0.00109524 | 3.26 ± 0.31 | 2.81 ± 0.35 | 3.16 ± 0.32 | p < 0.001 |
| 27 | **volume left ventral dicencephalon** | 0.00107143 | 3.23 ± 0.34 | 2.79 ± 0.37 | 3.09 ± 0.31 | p < 0.001 |
| 28 | **standard deviation of white matter in right posterior cingulate gyrus** | 0.00102381 | 0.14 ± 0.01 (33) | 0.13 ± 0.01 (5) | 0.13 ± 0.01 (3) | p < 0.001 |
| 29 | **volume medulla oblongata** | 0.001 | 4.22 ± 0.34 | 3.95 ± 0.27 | 3.85 ± 0.32 | p < 0.001 |
| 30 | **sum of volume of bilateral putamen** | 0.00088095 | 7.25 ± 1.55 | 6.63 ± 0.82 | 6.17 ± 1.31 | 0.001 |

**Supplementary table s3:** **Approach 2 using imaging derived variables only – Variable Importance** of the 30 most discriminative variables. For numeric variables, the significance of the diagnosis being discriminatory is assessed using linear (numeric variables) or logistic (categorial variables) regression. Volumes are presented as volume of the ROI as fraction of the total intracranial volume [‰] Cortical thickness is presented in mm. Values are presented as mean ± sd If missing values occur in a variable, their number is stated in parenthesis. If missing values occur in a variable, their number is stated in parenthesis.

**Literature**

[1] AW, Pike GB, Neto PR, Evans A, Zhang J, Huang H, Miller MI, van Zijl P, Mazziotta J (2008) Stereotaxic white matter atlas based on diffusion tensor imaging in an ICBM template. *Neuroimage* **40**, 570-582.

[2] Destrieux C, Fischl B, Dale A, Halgren E (2010) Automatic parcellation of human cortical gyri and sulci using standard anatomical nomenclature. *Neuroimage* **53**, 1-15.

[3] Fischl B, Salat DH, Busa E, Albert M, Dieterich M, Haselgrove C, van der Kouwe A, Killiany R, Kennedy D, Klaveness S, Montillo A, Makris N, Rosen B, Dale AM (2002) Whole Brain Segmentation: Automated Labeling of Neuroanatomical Structures in the Human Brain. *Neuron* **33**, 341-355.

[4] Iglesias JE, Van Leemput K, Bhatt P, Casillas C, Dutt S, Schuff N, Truran-Sacrey D, Boxer A, Fischl B (2015) Bayesian segmentation of brainstem structures in MRI. *Neuroimage* **113**, 184-195.
